# Unraveling the Molecular Mechanisms by Which the miR171b-*SCL6* Module Regulates Maturation in *Lilium*

**DOI:** 10.3390/ijms25179156

**Published:** 2024-08-23

**Authors:** Qing Li, Meiqi Song, Yachen Wang, Ping Lu, Wei Ge, Kezhong Zhang

**Affiliations:** 1 College of Landscape Architecture, Beijing University of Agriculture, Beijing 102206, China; 202130512019@bua.edu.cn (Q.L.); 202230512013@bua.edu.cn (M.S.); 202230527119@bua.edu.cn (Y.W.); 202330512021@bua.edu.cn (P.L.); 2 Beijing Laboratory of Urban and Rural Ecological Environment, Beijing 102206, China

**Keywords:** *Lilium*, phase transition, molecular mechanisms, *Lbr*-miR171b, *LbrSCL6*

## Abstract

*Lilium* is one of the most widely cultivated ornamental bulbous plants in the world. Although research has shown that variable temperature treatments can accelerate the development process from vegetative to reproductive growth in *Lilium*, the molecular regulation mechanisms of this development are not clear. In this study, *Lbr*-miR171b and its target gene, *LbrSCL6*, were selected and validated using transgenic functional verification, subcellular localization, and transcriptional activation. This study also investigated the differential expression of *Lbr*-miR171b and *LbrSCL6* in two temperature treatment groups (25 °C and 15 °C). *Lbr*-miR171b expression significantly increased after the temperature change, whereas that of *LbrSCL6* exhibited the opposite trend. Through in situ hybridization experiments facilitated by the design of hybridization probes targeting *LbrSCL6*, a reduction in *LbrSCL6* expression was detected following variable temperature treatment at 15 °C. The transgenic overexpression of *Lbr*-miR171b in plants promoted the phase transition, while *LbrSCL6* overexpression induced a delay in the phase transition. In addition, LbrWOX4 interacted with LbrSCL6 in yeast two-hybrid and bimolecular fluorescence complementation assays. In conclusion, these results explain the molecular regulatory mechanisms governing the phase transition in *Lilium*.

## 1. Introduction

*Lilium* is a perennial bulbous plant in the Liliaceae family that occupies an important position in the global flower industry due to its long history of cultivation, ornamental use, and application, as well as its beautiful and fragrant flowers [1]. In the past few years, the planting area of OT *Lilium* (a hybrid of Oriental *Lilium* × Trumpet *Lilium*) has swiftly expanded in China, especially that of red ‘Robina’, which is popular among consumers because of its robust stalks, large and colorful flowers, long ornamental period, and strong disease resistance, all of which make it highly desirable for *Lilium* industry development. Currently, the main problem affecting *Lilium* cultivation is its long juvenile period. Under natural conditions, *Lilium* cuttings or small bud cores bred by histoculture accumulate slowly in the first year and grow only one leaf, and the growth of the main stem into adulthood accelerates assimilate accumulation in the bulb, making the bulb expand rapidly [2]. Small *Lilium* bulblets require a minimum of 3 years to develop into large commercial bulbs, leading to a high bulb breeding cost.

Higher plant growth is a complex process involving many developmental stages in the life cycle that are classified by morphological characteristics and variability in organ development [3]. Plant growth phases can be divided into three stages: early developmental stage, mature nutritional stage, and reproductive stage [4]. The juvenile to mature transformation phase is known as the vegetative growth phase transition (VPC), whereas the mature to reproductive transformation phase is known as the reproductive growth phase [5]. The sequential transitional shifts of plants between these phases are also known as the phase transitions of plant growth [3,6].

At present, research on *Lilium* has mainly focused on floral organ development, although a few studies have investigated the regulation mechanism of phase transitions. Variable temperature treatment can induce phase transition in juvenile *Lilium* bulbs, thus shortening the juvenile stage and accelerating the advance into the adult stage; however, the molecular regulatory mechanism of this process needs to be further investigated. miRNAs represent a class of naturally generated, small endogenous noncoding RNA molecules, which are about 21–25 nucleotides long, highly conserved, temporal, and tissue specific [7]. Studies on plant miRNAs have shown that miRNAs generally achieve perfect pairing with the mRNA of target genes and then complete the negative regulation mechanism by cleaving the mRNA sequences of the target genes and degrading them [8]. miRNAs regulate plant auxin signal transduction and metabolism, flowering time regulation, flower and root development, phase transition during plant growth and development, and adversity and stress responses [9]. The main regulatory factors involved in the phase transition process of plant growth and development include miR156, miR159, miR171, and miR172 [10,11]. miR171 is an ancient and conserved miRNA family whose members are 21 nucleotides long. This family is widespread in plants, spanning bryophytes, monocots, and dicots [12]. The number of mature miR171s varies significantly among species. For example, *Hordeum vulgare*, *Brachypodium*, *Oryza sativa*, and *Zea mays* have 2, 4, 9, and 14 miR171s, respectively [13].

The roles and functions of miR171 have gradually been investigated in recent years, and miR171s are expressed in various plant organs [14]. Fan et al. [10] showed that *Osa-miR171*c mediated four *OsHAM* transcription factors to regulate the transition from vegetative to reproductive growth homeostasis in *Oryza sativa*. Li et al. [15] recently showed that miR171 was involved in the whole growth phase transition process. The miR171 family has been identified in plants through the downregulation of target gene *SCARECROW-LIKE6* (*SCL6*), which plays a role in the processes involved in branch tillering, meristematic tissue maintenance, root growth, leaf formation, and somatic embryogenesis in plants [10,16].

The SCL6 protein, which belongs to the HAM branch of the GRAS transcription factor family, plays a significant role in the maintenance of the undifferentiated state of plant shoot apical meristem (SAM) cells, the transformation of reproductive growth, and the response to adversity stress [17]. GRAS transcription factors are plant-specific transcription factors with functions such as GA responses, flowering time control, and apical meristem development [10]. Llave et al. [18] isolated miR171 in *Arabidopsis thaliana* for the first time and found that miR171 targets three *SCL6* genes, namely, *SCL6-II*, *SCL6-III*, and *SCL6-IV*, also known as *HAIRY MERISTEM* (*HAM*) and *LOST MERISTEMS* (*LOM*). Shan et al. [19] verified this, showing that the expressed structural domains of miR171 family members overlapped with the mRNA sequence of *SCL6-II/III*. miR171 and its target gene, *SCL6*, have been demonstrated to play important regulatory roles in plant vegetative and reproductive growth [20]. Li et al. [15] found that miR171b (*OX*171b)-overexpressing *Oryza sativa* plants showed increased plant rice blast resistance and regulated flowering time by suppressing *SCL6-IIs* expression. Curaba et al. [21] showed that miR171 acted by downregulating *SCL6-II/III/IV* genes in *Hordeum vulgare*, thereby participating in the juvenile to adult transition. Hai et al. [22] inferred that *Pinus densata* miR171 and its target gene *SCL6* had important regulatory roles in the early stages of root growth, leaf formation, and flowering. The relationship between *Lbr*-miR171 and its target gene, *LbrSCL*6, is not an independent regulatory pathway, as numerous factors are involved in the plant phase transition. Thus, it can be reasonably speculated that proteins interacting with the *LbrSCL6* transcription factor also exist in *Lilium*, collectively advancing the phase transition process.

miR171 is not independently regulated in relation to its target gene *SCL6*, and many other factors are involved in the plant phase transition process. Transcription factor WUSCHEL-RELATED HOMEOBOX4 (WOX4) is expressed in meristematic tissues during the phase transition in *A*. *thaliana* and is a key regulator controlling the stem cell population in shoot and root tips [23]. Zhou et al. [24] demonstrated that WOX4, WOX5, and HAM family proteins interact with each other to jointly advance the proliferation of plant stem cells, which is important for promoting SAM and root apical meristem (RAM) tissue development. Therefore, it is reasonable to hypothesize that proteins interacting with SCL6 transcription factors in *Lilium* jointly promote the phase transition process.

Most studies have focused on the regulatory role of miR171 and its target genes in the development of various organs, and there are few reports on its role in the transition from vegetative to reproductive growth in plants. To explore the molecular mechanisms underlying the effects of variable temperature treatment on the juvenile to adult transition in *Lilium* bulbs, this study used variable temperature-treated *Lilium* ‘Robina’ bulbs, from which the gene cloning, transgenic function, and biochemical characterization of *Lilium Lbr*-miR171b and its target gene *LbrSCL6* and the interactions between LbrWOX4 and LbrSCL6 were verified, revealing the regulatory mechanisms in the *Lilium* phase transition. This study provides an important scientific basis for shortening the growth cycle in *Lilium* and accelerating *Lilium* industry development.

## 2. Results

### 2.1. Bioinformatics Analysis of Lbr-miR171b and LbrSCL6

**Figure 1:**
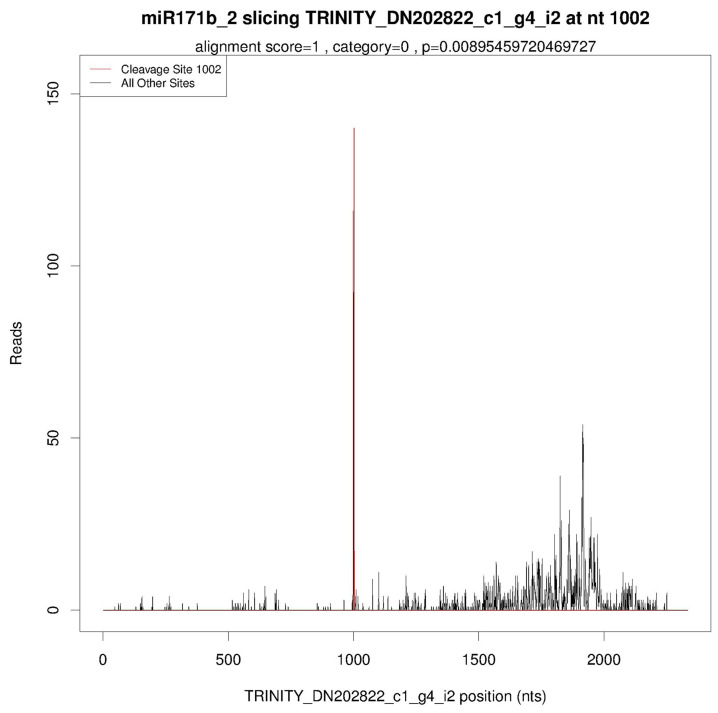
T-plot graph of *Lbr*-miR171b and its target gene *LbrSCL6*. Under CK treatment, *Lbr*-miR171b targets *LbrSCL6*. The vertical axis of the T-plot represents the number of reads at a specific cleavage site, indicating the quantity of degradation fragments with identical sequences at this position. The horizontal axis provides positional information on the mRNA sequence located within the transcript. The red line represents the predicted positive miRNA cleavage sites.

Using the degradation profile data of *Lilium* bud cores, a T-Plot graph was constructed, revealing a cleavage site at the 1002 nt position, demonstrating the existence of a targeted cleavage relationship between *Lbr*-miR171b and *LbrSCL6* (Figure 1).

The heatmap demonstrated that the expression trend was divided into two groups: an upregulation trend and a downregulation trend (Figure S1). The expression abundance of *Lbr*-miR171b in *Lilium* bud cores increased after 15 °C variable temperature treatment, whereas the corresponding target gene *LbrSCL6* showed reduced expression.

**Figure 2:**
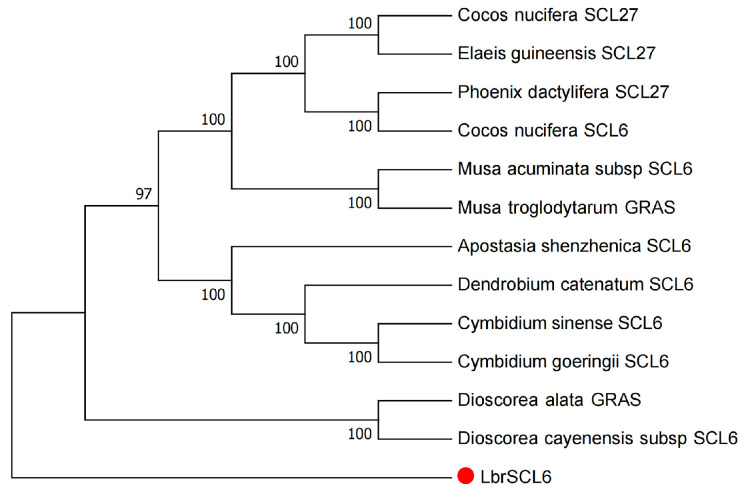
Phylogenetic analysis of LbrSCL6. The phylogenetic tree was constructed using the neighbor-joining (NJ) technique.

In the NCBI database, GRAS family proteins with relatively high similarity were identified through alignment. Using MEGA 7.0, the amino acid sequence of the LbrSCL6 protein was used to construct a phylogenetic tree with *Dioscorea cayenensis*, *Musa acuminata*, *Cocos nucifera*, and other species (Figure 2).

Conserved motif analysis of the LbrSCL6 protein showed that the protein structure of the LbrSCL6 transcription factor contained a highly conserved GRAS structural domain (motifs 1, 4, and 6) at the C-terminal end, demonstrating that it belongs to the GRAS family. Moreover, LbrSCL6 contained motifs 3 and 11 at the N terminus and motifs 12 and 20 at the C terminus (Figure S2).

### 2.2. Validation of qRT-PCR for Lbr-miR171b and LbrSCL6

The expression of *Lbr*-miR171b and its corresponding target gene, *LbrSCL6*, which was linked to the VPC of *Lilium*, was verified by qRT-PCR. *Lbr*-miR171b exhibited noticeable upregulated expression, whereas *LbrSCL6* displayed marked downregulated expression (Figure S3). The expression profiles of *Lbr*-miR171b and *LbrSCL6* exhibited inverse patterns, which were in line with the bioinformatics analysis. *Lbr*-miR171 was further demonstrated to negatively regulate *LbrSCL6*.

### 2.3. Identification of the Targeting Relationship between Lbr-miR171b and LbrSCL6

**Figure 3:**
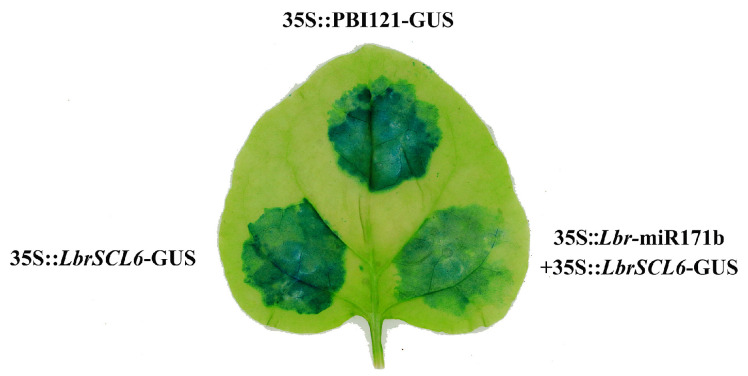
Validation of the interaction between *Lbr*-miR171b and *LbrSCL6*. The pBI121 empty vector was the control. The blue color represents GUS signaling.

GUS staining revealed that when the *N*. *benthamiana* leaves were injected separately with the pBI121 empty vector and 35S::*LbrSCL6*-GUS, the color was blue, with a significant increase in the GUS signal, whereas the GUS signal was significantly reduced in the leaves co-injected with a mixture of 35S::miR171b + 35S::*LbrSCL6*-GUS (Figure 3). These results suggest that *Lbr*-miR171b targets *LbrSCL6*.

### 2.4. Subcellular Localization and Transcriptional Activation of LbrSCL6 and LbrWOX4

**Figure 4:**
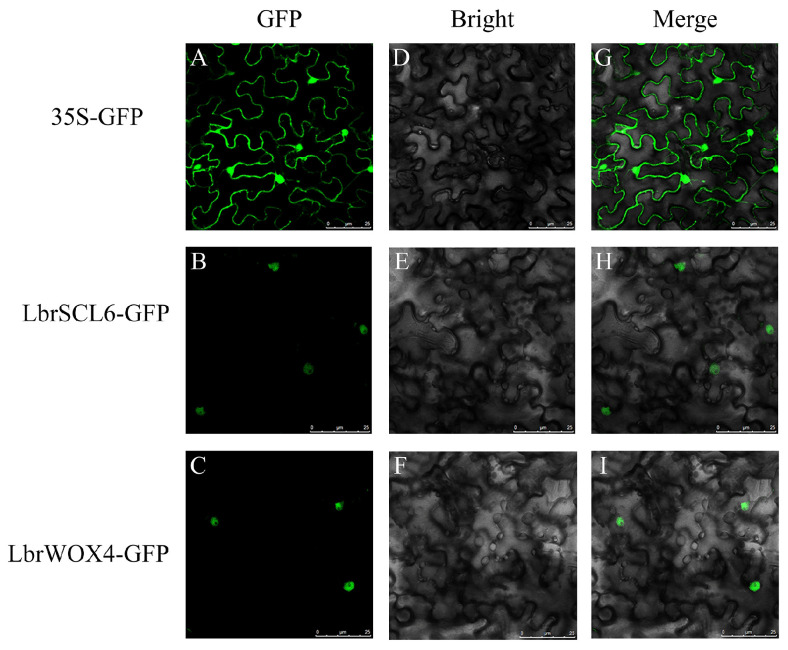
Subcellular localization of LbrSCL6 and LbrWOX4. (**A**–**C**) GFP fluorescent field. (**D**–**F**) Bright field. (**G**–**I**) Merged field. A 35S-GFP empty vector was used as a positive control. Scale bar = 25 µm.

Subcellular localization showed that the GFP signals of LbrSCL6 and LbrWOX4 were specifically localized in the nucleus, while the GFP of the empty vector was uniformly distributed throughout the cells (Figure 4).

Figure S4 demonstrates the results of the transcriptional activity analysis performed in the yeast system. In SD/-Trp-deficient medium, pNC-GBKT7 (BD), BD-LbrSCL6, and BD-LbrWOX4 grew normally, indicating that both the empty vectors and recombined plasmids were transferred into yeast strain AH109. In double-deficient solid medium SD/-Trp/-His, the final selection showed that BD-LbrSCL6 grew single colonies in 20 mmol/L 3-AT. BD-LbrWOX4 grew single colonies in 15 mmol/L 3-AT, and no colonies grew in negative control BD, suggesting that LbrSCL6 and LbrWOX4 induced His reporter gene expression to promote yeast growth. After adding X-α-Gal, the yeast colonies of BD-LbrSCL6 and BD-LbrWOX4 cultivated on SD/-Trp/-His medium exhibited a blue color. In contrast, the negative control BD showed no significant change. This assay demonstrated that LbrSCL6 and LbrWOX4 had transcriptional activation activity as transcription factors.

### 2.5. In Situ Hybridization of LbrSCL6

In situ hybridization of *LbrSCL6* was conducted to investigate the spatiotemporal expression pattern of *LbrSCL6* in *Lilium* bud cores after variable temperature treatment. *LbrSCL6* expression was absent in the negative control, while hybridization signals of *LbrSCL6* were observed in both the CK and CT groups. However, the hybridization signal was significantly weakened in the CT group (Figure S5), suggesting that the gene expression abundance of *LbrSCL6* was downregulated following variable temperature treatment.

### 2.6. Transgenic Functional Validation of Lbr-miR171b and LbrSCL6

**Figure 5:**
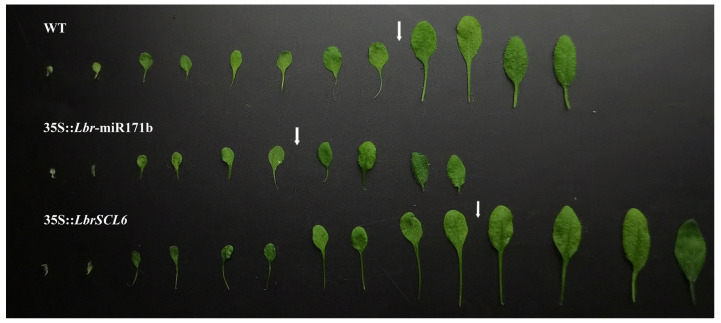
Rosette leaf morphology of *Lbr*-miR171b and *LbrSCL6* in transgenic *Arabidopsis thaliana*. The leaves depicted in the figure are organized based on their development sequence. The section to the right of the white arrow represents the beginning of epidermal hair on the distal axial surface, while the left part represents those that have not yet produced distal axial surface epidermal hair.

The transition from the juvenile to the adult phase in plants is symbolized by the appearance of epidermal hairs on the distal axial surface of the leaves. Transgenic *A*. *thaliana* was selected at the T3 generation. Wild-type (WT) *A*. *thaliana* initiated the development of epidermal trichomes on the distal axial surface starting at the ninth leaf (Figure 5). Conversely, *Lbr*-miR171b-overexpressing *A*. *thaliana* developed epidermal hairs on the distal axial surface as early as the seventh leaf, preceding the onset observed in WT plants. *LbrSCL6*-overexpressing *A*. *thaliana* developed epidermal hairs on the distal axial surface beginning at the 11th leaf. Thus, *Lbr*-miR171b promoted the juvenile to adult phase transition in plants, whereas *LbrSCL6* inhibited this process.

**Figure 6:**
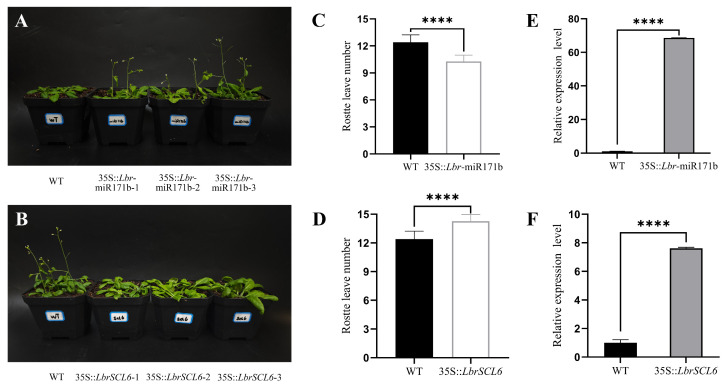
Overexpression results of *Lbr*-miR171b and *LbrSCL6* in *A. thaliana.* (**A**) Flowering phenotypes of WT and T3 generation 35S::*Lbr*-miR171b transgenic *A*. *thaliana*. (**B**) Flowering phenotypes of WT and T3 generation transgenic 35::*LbrSCL6 A*. *thaliana*. (**C**) Differential analysis of rosette leaf numbers between T3 transgenic *Lbr*-miR171b and WT plants. (**D**) Differential analysis of rosette leaf numbers between T3 transgenic *LbrSCL6* and WT plants. (**E**) Expression levels of *Lbr*-miR171b in WT and T3-generation transgenic plants. (**F**) Expression levels of *LbrSCL6* in WT and T3-generation transgenic plants. Significant differences are shown (**** *p* < 0.0001), *n* = 15.

By comparing flowering time and rosette leaf number in WT and transgenic *A*. *thaliana* plants, we found that *Lbr*-miR171b overexpression promoted earlier flowering and resulted in fewer rosette leaves at flowering (Figure 6C,D). By contrast, *LbrSCL6* overexpression resulted in delayed flowering time and more rosette leaves at flowering (Figure 6A,B). Furthermore, the expression of *Lbr*-miR171b and *LbrSCL6* in transgenic *A*. *thaliana* was significantly elevated compared with WT, as shown by qRT-PCR (Figure 6E,F).

### 2.7. Bimolecular Fluorescence Complementation of LbrSCL6 and LbrWOX4

**Figure 7:**
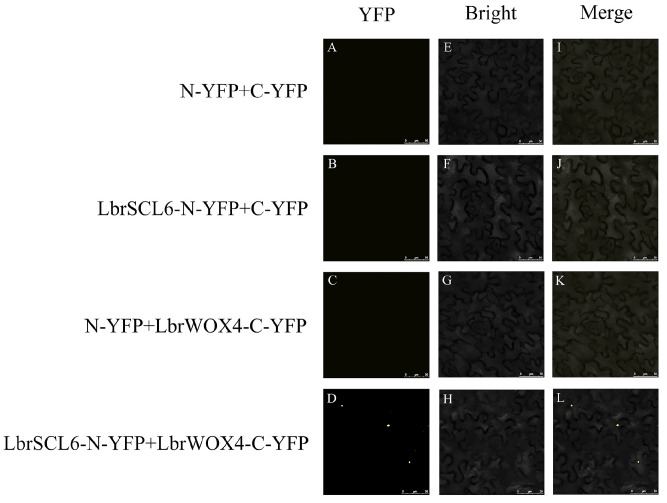
Bimolecular fluorescence complementation of LbrSCL6 with LbrWOX4. (**A**–**D**) YFP fluorescence field. (**E**–**H**) Bright field. (**I**–**L**): Merged field. Scale bar = 50 µm.

The lower epidermis of *N*. *benthamiana* injected with the infestation solution was placed under a confocal microscope to observe the YFP signal. No yellow fluorescence signal was detected in the lower epidermis of *N*. *benthamiana* in the control group (Figure 7). By contrast, in the experimental combination of LbrSCL6 and LbrWOX4, YFP was detected in the nucleus. LbrSCL6 interacted with LbrWOX4, and the interaction site was located in the nucleus.

### 2.8. Yeast Two-Hybridization of LbrSCL6 and LbrWOX4

**Figure 8:**
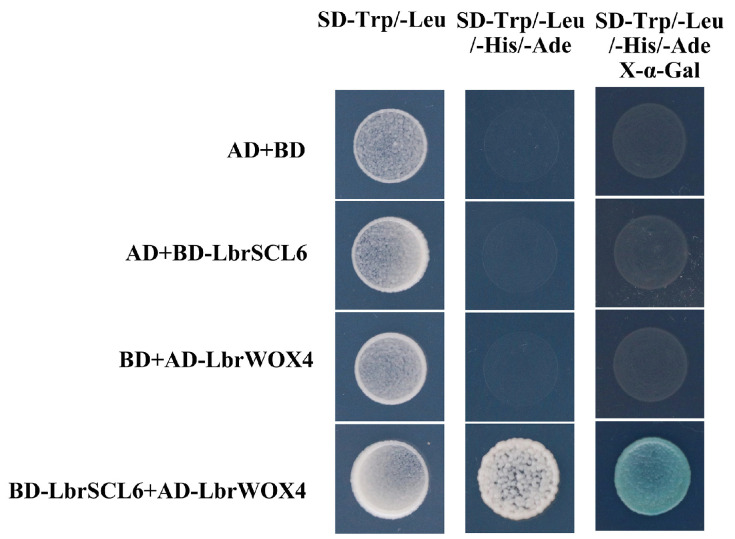
Yeast two-hybridization of LbrSCL6 and LbrWOX4. Empty vectors BD-pGBKT7 and AD-pGADT7 were used as negative controls.

Negative controls pNC-GADT7 + pNC-GBKDT7, pNC-GADT7 + pNC-GBKDT7-LbrSCL6, pNC-GADT7-LbrWOX4 + pNC-GBKDT7, and pNC-GADT7-LbrWOX4 + pNC-GBKDT7-LbrSCL6 were co-transformed into yeast strain AH109, and the bacterial fluids were added dropwise to double-deficient SD-Trp/-Leu and quadruple-deficient SD-Ade/-His/-Leu/-Trp solid medium. The yeast cells produced white colonies on the double-deficient medium, while only yeast cells containing pNC-GADT7-LbrWOX4 and pNC-GBKDT7-LbrSCL6 produced blue colonies on the quadruple-deficient medium (Figure 8), further illustrating that LbrSCL6 interacted with LbrWOX4.

## 3. Discussion

Researchers have found that variable temperature treatments affect the growth and development of *Lilium* bulbs, inducing the phase transition earlier and shortening the *Lilium* growth cycle [25]. miRNAs have a significant influence on plant growth and development by modulating the expression of corresponding target genes [26]. To investigate the molecular regulatory mechanisms in *Lilium* following variable temperature treatments, a series of investigations were conducted on *Lbr*-miR171b and its corresponding target gene *LbrSCL6*.

In the present study, the essential regulatory role played by *Lbr*-miR171b in the phase transition process of *Lilium* was demonstrated during temperature variation. The phase transition in plants represents a vital period of growth and development, closely associated with disease resistance, yield, and other agronomic traits. Previous research has indicated that phase transition is regulated by the expression of various genes [27]. miR171 was one of the earliest members discovered in the miRNA family of plants, participating in all plants’ growth and development processes and playing a crucial role [28]. In *Oryza sativa*, miR171b expression in leaves increases gradually with seedling growth. During the vegetative growth stage, the expression level of miR171b is low in the juvenile phase but increases in the adult phase [10]. miR171 overexpression in plant species can activate the miR156 pathway and inhibit the expression of *TRD* and *HvPLA1* genes, thereby altering the trophic growth time-phase transition in plants [21]. Some studies have demonstrated that miR171 can promote the transition from vegetative growth to reproductive growth in plants. Hai et al. [22] compared *pre*-miR171-overexpressing transgenic *Pinus densata* with WT plants and showed larger leaves and earlier flowering under long-day conditions. Zhu et al. [29] found that *Csa*-miR171a overexpression in *A. thaliana* increased the lateral root number and advanced the shoot extraction time. The regulatory role of miR171 in phase transition may differ depending on plant taxonomy. The specific effects and mechanisms of miR171 can vary significantly among plant species, reflecting its diverse functional adaptations across plant lineages [10,15,30].

Previous research has shown that miR171 targets three members of the GRAS family, *SCL6/SCL6-IV*, *SCL22/SCL6-III*, and *SCL27/SCL6-II*, which play important roles in processes such as cell proliferation in meristematic tissues, chlorophyll synthesis, floral organ development, and floral timing regulation [31]. By subjecting *Lilium* bud cores to alternating temperature treatments, we determined the targeting cleavage relationship between *Lbr*-miR171b and its target gene, *LbrSCL6*. In addition, the expression abundance of *Lbr*-miR171b increased after variable temperature treatment, while the expression abundance of the target gene, *LbrSCL6*, decreased according to clustering heatmap analysis of the CK and CT groups. It has been hypothesized that *LbrSCL6* plays a repressor role in the phase change in *Lilium*. Most GRAS family transcription factors contain a less conserved variable region in the N terminal and a conserved GRAS structural domain in the C terminal [32,33]. Phylogenetic tree analysis confirmed that LbrSCL6 clustered with other plants’ GRAS family proteins. Moreover, through bioinformatics analysis, we verified that the protein structure of LbrSCL6 had a remarkably conserved GRAS structural domain at the C terminus. We verified the expression of *Lbr*-miR171b and its target gene *LbrSCL6* in *Lilium* bud cores under different temperature treatments through qRT-PCR and found that the expression profiles of *Lbr*-miR171b and *LbrSCL6* were reversed, with significant upregulation of *Lbr*-miR171b and downregulation of *LbrSCL6*. Thus, we tentatively hypothesized that *Lbr*-miR171b and its target gene, *LbrSCL6*, exhibit complementary regulatory roles during the transition phase from vegetative to reproductive growth in *Lilium* bud cores. To more intuitively reflect this interaction, we used GUS staining and found that the GUS signal was significantly reduced after mixed injection of 35S::*Lbr*-miR171b+35::*LbrSCL6*-GUS compared with the injection of 35S::*LbrSCL6* alone, indicating that there was an inhibitory effect. Therefore, *Lbr*-miR171 targeted and repressed *LbrSCL6*.

To enrich the biochemical characterization of LbrSCL6, we selected a WOX protein that could interact with LbrSCL6 in *Lilium* using biosignature analysis and named it LbrWOX4 according to the genetic relationship. Transcription factors in the WOX family are important in regulating growth and developmental processes in eukaryotic organisms. Laux et al. [34] first identified the WUSCHEL (WUS) transcription factor in the model plant *A*. *thaliana* and confirmed its significant involvement in apical meristem development. In the past decade, attempts have been made to clone WUSCHEL-related transcription factors from different species, and they have all been shown to contain a conserved structural domain homeobox, which have since been formally named WOX transcription factors [35]. The specific function of individual WOX family proteins likely depends on their spatiotemporal expression patterns and possibly on their interactions with other proteins, which may inhibit their transcriptional activity [36]. Many studies have shown that WOX family members play important roles in the genesis and maintenance of undifferentiated cells in plants [37]. The negative feedback regulatory system of WUS and CLV3 serves to maintain the activity of the SAM, and several genes involved in this regulation together form the core of the autoregulation that maintains the activity in the phloem tissues at the stem tip [38]. The HAM subfamily of the GRAS family plays a vital role in preserving the undifferentiated state of the plant SAM [39]. Studies in plant species such as *Petunia* have shown that the HAM family dominates the autoregulatory signaling system, which, together with the WUS/CLV3 module, mediates the interaction between external environmental signals and the differentiation of the phloem tissue at the stem tip [40]. Moreover, He et al. [33] found that in *Lilium*, two WUS family transcription factors, LlWOX9 and LlWOX11, act as positive regulatory factors in the formation of *Lilium* bulbs. Therefore, it is reasonable to speculate that there is an interaction between the LbrSCL6 and LbrWOX4 proteins in *Lilium* that jointly advances the phase transition process.

Transcription factor proteins are primarily localized in the cell nucleus, where they exert transcriptional regulatory functions, with some localized in the cytoplasm, mitochondria, cell membrane, and other cellular compartments [41]. Subcellular localization showed that LbrSCL6 and LbrWOX4 localized separately in the nucleus, corresponding with the results of subcellular localization for SCL6 and WOX4 in *A*. *thaliana* [17,33]. This is because the transcription factors possess signaling and metabolic functions that guide the proteins into the nucleus to execute their functions [41]. The transcriptional activation results showed that both LbrSCL6 and LbrWOX4 exhibited transcriptional activation activity, indicating that both play important roles as transcription factors in the *Lilium* growth process.

Studying the temporal and spatial expression of plant genes is an important means to explore plant growth and development mechanism [42]. In situ hybridization, as a direct method for studying gene localization and spatiotemporal expression, is now widely used in growth and development studies of roots, stems, shoots, fruit, and other parts of plants [43]. To deeply examine the spatiotemporal expression pattern of *LbrSCL6*, we performed in situ hybridization experiments and showed that after the temperature change treatment, the hybridization signal of *LbrSCL6* significantly decreased, suggesting that *LbrSCL6* is involved in the process of phase transition regulation in *Lilium*. Moreover, LbrSCL6 is typically expressed at the position of the apical meristem and participates in the maintenance and differentiation of the apical meristem [39]. In the results, the hybridization signals are mainly concentrated at the apex of the growth cone, which verifies this point.

In transgenic functional analysis, qRT-PCR validation was performed on T3-generation transgenic plants. Compared with WT, the expression levels were elevated in transgenic plants, indicating the successful construction of *Lbr*-miR171b- and *LbrSCL6*-overexpressing plants. In phase transition studies conducted in the model plant *A*. *thaliana*, the transition from juvenile to adult is marked by changes in leaves from rounded to spatulate and leaf edges from smooth to serrated and the appearance of epidermal white tomentum at the distal axial end of the leaves, and the sign of its transition from the adult phase to the reproductive phase is rosette leaf abscission [44]. By observing and recording this phenotype, we found that transgenic *Lbr*-miR171b-overexpressing plants produced distal leaf abaxial hairs earlier, formed fewer rosette leaves at flowering, had an advanced flowering time, and entered the reproductive stage earlier than WT *A. thaliana*. Consequently, *Lbr*-miR171b promotes the promote the process of temporal phase transition from juvenile to reproductive stages in *Lilium*. By contrast, *LbrSCL6*-expressing transgenic lines showed a later appearance of epidermal hairs on distal leaves, more rosette leaves at flowering, and delayed flowering, suggesting that *LbrSCL6* inhibits the temporal phase transition in *Lilium*. Overexpression of miR171b, *SCL6*, and their orthologs in multiple species has been shown to result in similar phenotypic effects. Huang et al. [45] discovered that *SlGRAS24* in *Solanum lycopersicum* was an *AtSCL6* ortholog and a target gene of *Sly*-miR171. Compared with WT plants, *Sly*-miR171-OE plants exhibited an accelerated transition from vegetative to reproductive development, leading to earlier flowering, whereas *SlGRAS24*-OE plants showed a delayed phase transition and significantly postponed flowering time [46]. Xue et al. [47] found that miR171 regulates three *LOM* genes, namely, *LOM1*, *LOM2*, and *LOM3* (also referred to as *AtHAM1*, *AtHAM2*, and *AtHAM3*, or *SCL6-2*, *SCL6-3*, and *SCL6-4,* respectively) in *A. thaliana*. Among these, *LOM1* overexpression under long-day conditions results in delayed flowering time [47]. Xing et al. [48] investigated the miR171-*SCL6* module and found that overexpression of the *LaSCL6* gene from of *Larix gmelinii* prolongs the juvenile phase in *A. thalian*a, delaying bolting and flowering times compared with WT *A. thaliana*, indicating that *LaSCL6* overexpression inhibits the transition from vegetative to reproductive growth. We hypothesized that transgenic *A*. *thaliana* exhibited earlier flowering and a decreased count of rosette leaves due to the induction of *A*. *thaliana* by *Lbr*-miR171b to utilize fewer assimilates during the vegetative growth phase, thereby potentially allocating more resources to the reproductive growth phase. Therefore, it is hypothesized that the fluctuation of miR171 levels during the phase transition may be an effective method for improving yield providing new ideas for plant breeding [27].

Zhou et al. [24] demonstrated that an interaction occurred between the WOX4 protein and the HAM protein in *A*. *thaliana* and collectively promoted the proliferation of plant stem cells, which was crucial for the development of the SAM and RAM. To determine whether a similar interaction existed between LbrSCL6 and LbrWOX4 proteins in *Lilium*, we used a bimolecular fluorescence complementation (BIFC) assay for validation. In the control group, no yellow fluorescent signal was detected in the lower epidermis of *N*. *benthamiana*. By contrast, YFP was detected in the nuclei of multiple cells in the experimental combination of LbrSCL6 and LbrWOX4. Therefore, LbrSCL6 interacted with the LbrWOX4 protein, and the site of interaction was located in the nucleus.

Fields and Song [49] established the novel genetic approach known as the yeast two-hybrid system, which allows for the direct detection of protein–protein interactions. This technique analyzes the interaction between bait and prey proteins by observing the expression of reporter genes [50]. The yeast two-hybrid experiment showed that the mixture of both on a quadruple dropout medium activated yeast β-galactosidase to produce a blue signal, further confirming the interaction between LbrSCL6 and LbrWOX4 proteins and their collaborative role during the *Lilium* phase transition. Future studies should explore the specific mechanism of action in more detail.

Previous research has shown that the timing of phase transition in plants is tightly controlled by a complex network of gene regulation, and many genes involved in regulatory pathways have been identified in response to different external environments and endogenous factors. We successfully demonstrated that *Lbr*-miR171b in *Lilium* inhibited the expression of its target gene *LbrSCL6* and found that *Lbr*-miR171b overexpression promoted the transition from the juvenile phase to the reproductive phase. *LbrSCL6* overexpression inhibited this process, indicating their involvement in the relevant regulatory pathways of the *Lilium* phase transition. Furthermore, this study revealed the interaction between LbrSCL6 and LbrWOX4, although without exploring specific developmental stages. These findings contribute to the further exploration of the molecular mechanisms underlying the phase transition in *Lilium* bulbs, accelerating bulb maturation processes and offering new insights for future research endeavors.

## 4. Materials and Methods

### 4.1. Plant Material

The cutting materials used in this experiment were obtained from the Oriental × Trumpet *Lilium* hybrid variety ‘Robina’. We selected bulbs with a 14–16 cm circumference, uniform size, and no pests or mechanical damage. Bulblets were obtained by excising the outer layers of *Lilium* scales within a pre-sterilized substrate (peat/vermiculite = 3:1). The experiment was conducted with control (CK) and treatment (CT) groups. The CK group was cultivated at a constant temperature of 25 °C for 16 weeks and subsequently cultivated at 4 °C for 10 weeks. The CT group was cultivated at 25 °C for 12 weeks, followed by a 4-week period at 15 °C and a subsequent 10-week cultivation period at 4 °C. All processes were carried out in the dark. Bulblets of similar size were selected, and after rinsing with deionized water, the bud core was detached, immersed in liquid nitrogen, and stored at −80 °C.

### 4.2. Bioinformatics Analysis

Based on the FPKM transcriptome data previously analyzed by our research group, we selected the gene expression information of members belonging to the GRAS gene family and used TBtools (v1.108) software to cluster the data and construct a heatmap of gene expression. The parameters for analysis in TBtools were as follows: Width = 500, Height = 555, Scalemethod = Normalized; selecting ClusterRows and ClusterCols to cluster the values of rows and columns; selecting ColScale to normalize the data, Tileshape = Rectangle; DistMethod = Euclidean, ClusterMethod = Complete. Using MAGA 7.0 software, the family history of the LbrSCL6 transcription factor cluster and GRAS family in *A*. *thaliana*, *Musa acuminata*, *Lilium*, and other species was inferred using the neighbor-joining (NJ) method. The assessment criterion of p-distance was applied to compute the evolutionary divergence, enabling the construction of systematic phylogenetic trees. The MEME online program was utilized to conduct protein motif analysis for LbrSCL6. MEME analysis was conducted locally using the following settings: a maximum of 20 motifs allowed, number of repetitions set to any, minimum motif width set to 6, and maximum motif width set to 200. Utilizing degradation profile data from *Lilium* bud cores collected by our research team, T-Plot diagrams were generated to illustrate the relationship between miRNAs and their corresponding transcript cleavage sites.

### 4.3. qRT-PCR Analysis

The relative expression levels of *Lbr*-miR171b and its corresponding target gene *LbrSCL6* were verified using real-time fluorescence quantitative PCR (qRT-PCR). Total RNA was isolated from *Lilium* ‘Robina’ bud cores in CK and CT groups using a MolPure^®^ Plant Plus RNA Kit (Aidlab, Beijing, China). The first-strand cDNA synthesis for *LbrSCL6* fluorescence quantification was completed using the TransScript^®^ One-Step gDNA Removal and cDNA Synthesis SuperMix Kit (TransGen, Beijing, China), and the TransScript miRNA First-Stand cDNA Synthesis SuperMix Kit (TransGen) was used for *Lbr*-miR171b fluorescence quantification. Primers were designed using the online Primer3Plus software (version 0.4.0) (Table S1). The enzyme employed in qRT-PCR in this experiment was 2× SYBR Green qPCR Mix (Aibosen, Beijing, China). The qRT-PCR reaction proceeded with the following steps: 95 °C for 2 min, followed by 50 cycles of 95 °C for 15 s, 57 °C for 15 s, and 72 °C for 20 s. There were three biological replicates, and the mean values were calculated. The relative expression levels in the samples were determined using the 2^−ΔΔCt^ method, and significance analysis was conducted. U6 snRNA was used as the internal reference gene for *Lbr*-miR171b [51], and 18S rRNA was used as the internal reference gene for *LbrSCL6* [52]. The primers employed in qRT-PCR are listed in Table S1.

### 4.4. Gene Cloning

The extraction of total DNA from *Lilium* bud cores was accomplished using a Plant Genomic DNA Extraction Kit (CTAB; Aidlab, Beijing, China). In accordance with the predicted *Lbr*-miR171b precursor sequence information, primers were designed using the Primer3Plus online tool, and DNA was used as a PCR amplification template. The product was transferred into the pTOPO-T vector to complete the sequence comparison. *LbrSCL6* and *LbrWOX4* were cloned from the cDNA reverse-transcribed from the total RNA of the *Lilium* bud core.

### 4.5. Validation of the Targeting Relationship between Lbr-miR171b and LbrSCL6

Using a double enzyme digestion method, the PCR-cloned *Lbr*-miR171b precursor sequences and *LbrSCL6* complete coding sequences (CDSs) were constructed into pBI121 vectors. The successfully constructed pBI121-*LbrSCL6* and pBI121-*Lbr*-miR171b recombined vector plasmids and the empty carrier plasmid were transferred into *Agrobacterium* GV3101. Individual colonies were selected, cultured in YEB liquid medium supplemented with rifampicin (Rif) and kanamycin (Kan) with agitation for 1–2 days, adjusted to an OD600 of 0.6, and subsequently infiltrated into the abaxial surface of *Nicotiana benthamiana* leaves by zone injection. After infiltration, they were kept in the dark for 24 h and then cultivated with light for 48–72 h. *Nicotiana benthamiana* leaves were collected fully covered with the prepared GUS staining solution, soaked for 15 min, and subjected to vacuum infiltration until the solution completely penetrated the leaf tissue. The leaves were then incubated at 37 °C in the dark on a shaker for 18 h, decolorized with 75% ethanol for 4–8 h, and observed for changes in leaf color with the naked eye.

### 4.6. Subcellular Localization and Transcriptional Activation

Subcellular localization prediction of LbrSCL6 and LbrWOX4 was first performed using the online prediction software ProtComp 9.0. After the target gene plasmid was extracted, amplification was performed using primers supplemented with a seamless cloning universal junction. The amplified product was then connected to the enzymatically cleaved pNC-Cam1304-SubN vector (containing a GFP marker) [53]. The successfully constructed recombined plasmid and empty plasmid were transformed into *Agrobacterium tumefaciens* GV3101 in the sensory state using the freeze–thaw method to identify the positive clones. With the assistance of a subcellular localization infiltration solution, the vector plasmid was introduced into the lower epidermal cells of native *N*. *benthamiana* leaves, and the leaves were incubated with light for 1–2 days after dark treatment for 24 h. The lower epidermal layer of *N*. *benthamiana* leaves was cut and prepared, and the fluorescence signal was observed using a fluorescence microscope.

To investigate the transcriptional activation activities of LbrSCL6 and LbrWOX4, the *LbrSCL6* CDS was inserted into the pNC-GBKT7 vector, and pNC-GBKT7-LbrSCL6, pNC-GBKT7-LbrWOX4, and the empty vectors were introduced into yeast strain AH109 and incubated on SD/-Trp-deficient solid medium at 28 °C for 2 days. After dilution, the bacterial solution was incubated on SD/-Trp/-His-deficient solid medium. BD-LbrSCL6 corresponded to the addition of 20 mmol/L 3-AT, while BD-LbrWOX4 corresponded to the addition of 15 mmol/L 3-AT. After 2–3 days of incubation at 28 °C, 1 µL of X-α-Gal was supplemented, and staining was observed for identification.

### 4.7. In Situ Hybridization

*Lilium* bud cores from the CK and CT groups were retrieved from a −80 °C freezer, rinsed, and placed in fixation solution (aqueous preparation of DEPC) for at least 12 h. Dehydration was completed with different alcohol gradients, followed by sectioning. After deparaffinization and paraffin sections dehydration, digestion was completed by adding proteinase K dropwise at 37 °C. Subsequently, the tissue slices were incubated in a pre-hybridization buffer at 37 °C for 1 h. Upon removal of the buffer, a hybridization buffer containing 1 µM of the *LbrSCL6* probe was added and maintained at a constant temperature of 42 °C overnight. After hybridization, BCIP/NBT coloring solution was added, and the coloring reaction was observed under a microscope. After rinsing, the sections were sealed with glycerol gelatin, and the images were captured and analyzed.

### 4.8. Transgenic Functional Verification

The target genes were constructed in the overexpression vector pNC-Cam3304-MCS35S employing the Nimble cloning (NC) method [53], resulting in recombined pNC-Cam3304-MCS35S-*LbrSCL6* and pNC-Cam3304-MCS35S-miR171b. The recombined plasmids were transferred into *Agrobacterium* GV3101, and the bacterial solution was infiltrated into WT *A*. *thaliana* by inflorescence infestation. The inoculated plants were nurtured and selected until they reached the T3 generation. The morphology of epidermal hair on the distal axial surfaces and the flowering time were observed. The relative expression of *Lbr*-miR171b and *LbrSCL6* in transgenic T3-generation *A*. *thaliana* lines was verified using qRT-PCR. The primers employed in qRT-PCR are listed in Table S1.

### 4.9. Bimolecular Fluorescence Complementation and Yeast Two-Hybrid Assays

The CDSs of *LbrSCL6* and *LbrWOX4* were individually ligated into the pNC-BIFC-ENN (containing the NYFP tag sequence) and pNC-BIFC-ECN vectors (containing the CYFP tag sequence). The two constructed recombined vector plasmids and two empty vector plasmids (negative control) were transformed into *Agrobacterium* GV3101. As per the test combinations (Table S2), the infiltration solutions were mixed in equal proportions and infiltrated into the abaxial surface of *N*. *benthamiana* leaves using the transient transformation method and incubated in the dark for 24 h and with light for 1–2 days. Yellow fluorescent protein (YFP) signals in the lower epidermal cells of *N*. *benthamiana* leaves were observed using a laser confocal microscope (Leica TCS SP5, Wetzlar, Germany) at an excitation wavelength of 514 nm.

To further verify the interaction between these two genes, the CDSs of *LbrSCL6* and *LbrWOX4* were separately integrated into the pNC-GBKT7 and pNC-GADT7 vectors. The recombined vector plasmids, pNC-GADT7-LbrWOX4 and pNC-GBDT7-LbrSCL6, along with their corresponding empty vector plasmids, were mixed in proportion according to the experimental combinations listed in Table S3 and co-transformed into yeast strain AH109. The transformed yeast colonies were cultured on SD/-Trp-deficient medium. After confirming the accuracy of sequencing, the yeast colonies were diluted and spotted onto SD-Trp/Leu-deficient medium for initial selection. Afterward, they were diluted and spotted onto SD-Ade/-His/-Leu/-Trp-deficient medium for secondary selection, and after colony growth, 1 µL X-α-Gal was introduced for colorimetric assessment.

## 5. Conclusions

Based on bioinformatics analysis, we selected and identified *Lbr*-miR171b and its target gene, *LbrSCL6*, which are involved in the regulation of the *Lilium* phase transition. Gene cloning, qRT-PCR, GUS histochemical staining experiments, and transgenic functional validation demonstrated that *Lbr*-miR171b targets regulated *LbrSCL6*, with their functions exhibiting opposite outcomes. This suggests that *Lbr*-miR171b promotes the *Lilium* phase transition, while *LbrSCL6* inhibits this process. In situ hybridization verified the expression pattern of *LbrSCL6* and showed that LbrSCL6 expression was significantly decreased due to a temperature change. The biochemical characteristics of LbrSCL6 and LbrWOX4 were determined using subcellular localization and transcriptional activation assays. Both LbrSCL6 and LbrWOX4 were localized in the nucleus and possessed transcriptional activation activity. Subsequently, the LbrSCL6 protein interacted with the LbrWOX4 protein and localized in the nucleus, as shown using BiFC and yeast two-hybrid assays. This study provides a new scientific basis for shortening *Lilium* maturation and accelerating *Lilium* bulb culture.

## Data Availability

The datasets used and analyzed during the current study are available from the corresponding author upon reasonable request.

## Supplementary Materials

The following supporting information can be downloaded at https://www.mdpi.com/article/10.3390/ijms25179156/s1.
