# Supplementary material for: Unraveling the Molecular Mechanisms by Which the miR171b-SCL6 Module Regulates Maturation in Lilium
Source: Int J Mol Sci. 2024 Aug 23;25(17):9156. doi: 10.3390/ijms25179156 (PMC11394818; doi:10.3390/ijms25179156)
Supplement: Supplementary file 1 [file ijms-25-09156-s001.zip › ijms-3072295-supplementary.pdf]

Supplementary Materials

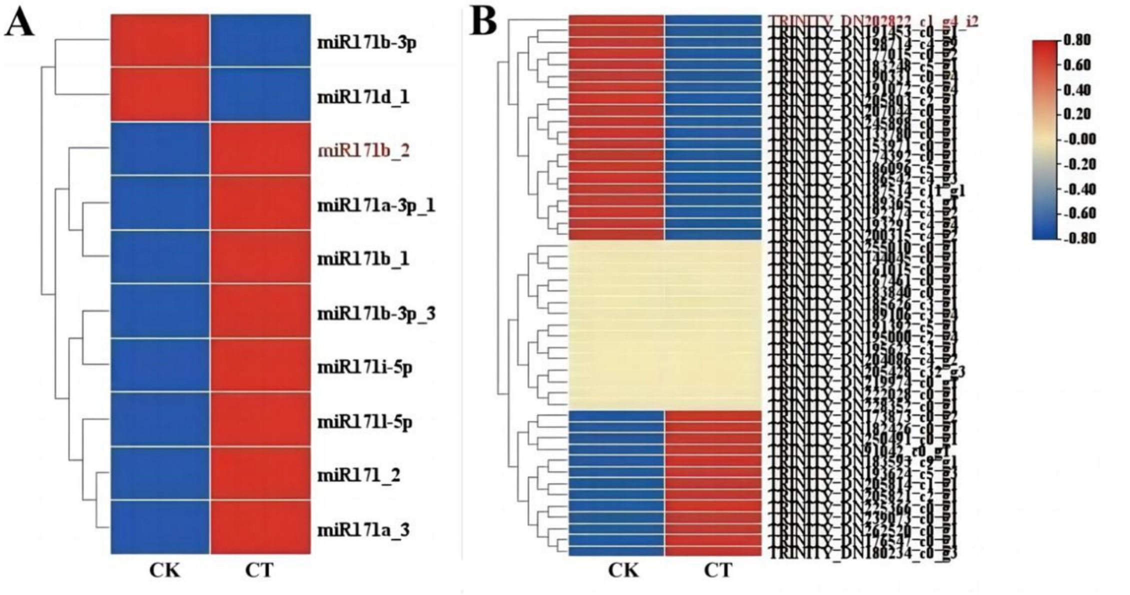

Figure S1. Differential expression of *Lbr*-miR171b and its target gene *LbrSCL6* in *Lilium* bud cores during variable temperature treatment. **(A)** The clustering heatmap of *Lbr*-miR171b. **(B)** GRAS family clustering heatmap of *Lilium*. Bars in the heatmap represent the range of gene expression levels log10 (FPKM + 1), and red and blue colors represent upregulated and downregulated expression, respectively.

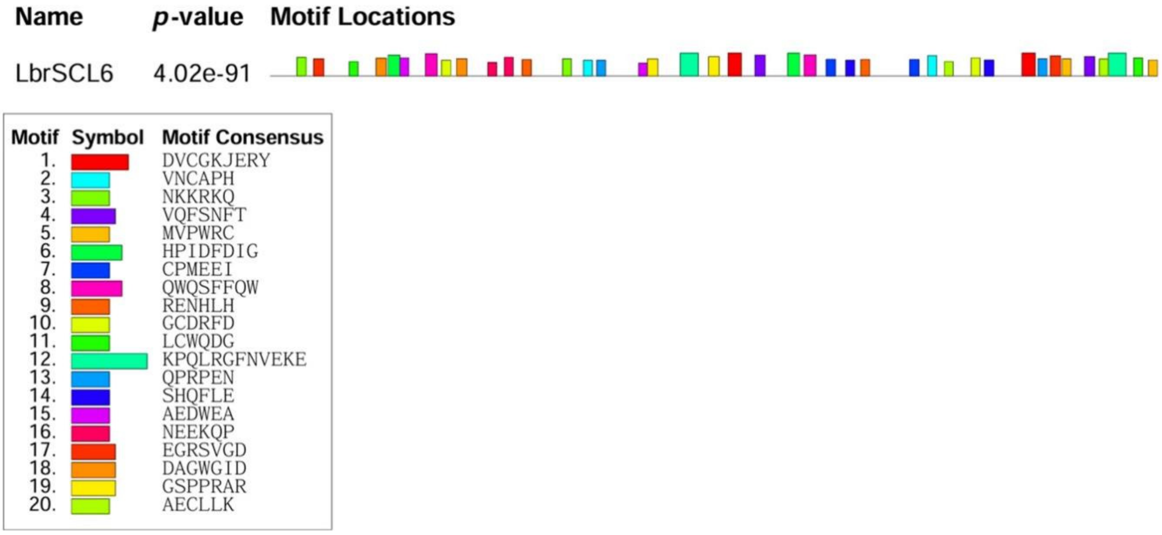

Figure S2. Protein module analysis of *LbrSCL6*. Each module is represented by colored boxes numbered 1–20.

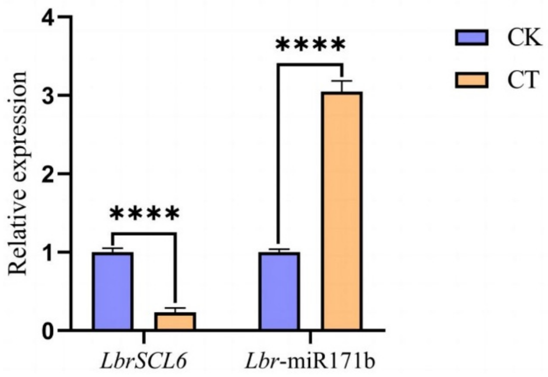

Figure S3. qRT-PCR results of *Lbr*-miR171b and *LbrSCL6*. Expression profiles of *Lbr*-miR171b and *LbrSCL6* in the CK and CT groups of *Lilium* bud cores are reflected. Expression of the CK group was normalized to 1.0. The bars illustrate the mean ± standard error derived from 3 replicates (*****P* < 0.0001).

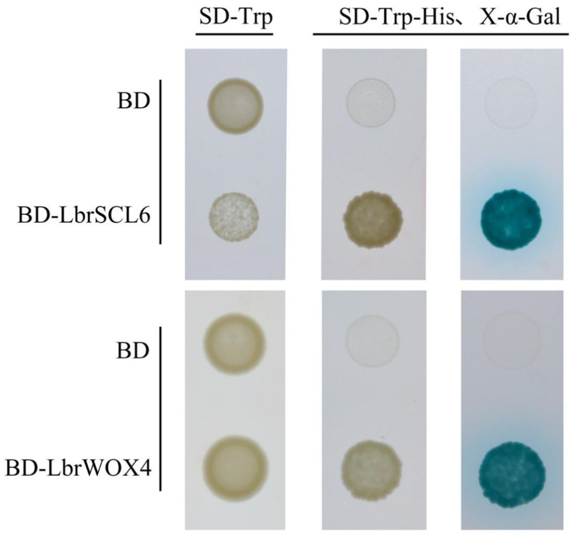

Figure S4. Transcriptional activation of LbrSCL6 and LbrWOX4. The blue signal indicates the activation of yeast β-Galactosidase.

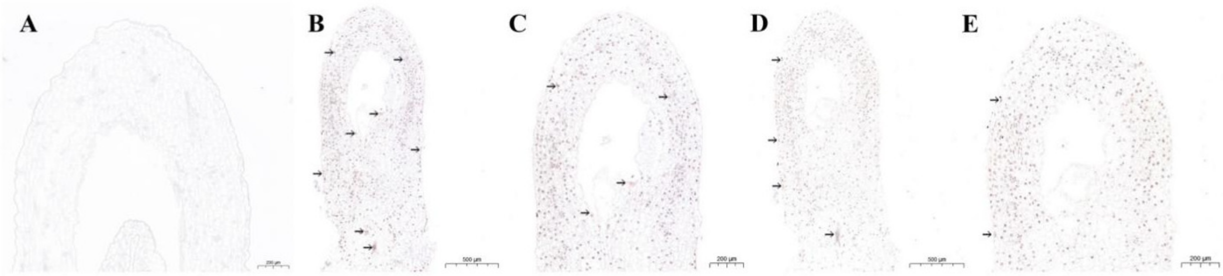

Figure S5. *In situ* hybridization results of *LbrSCL6*. **(A)** Negative control. **(B)–(C)** Expression of *LbrSCL6* in the CK group. **(D)–(E)** Expression of *LbrSCL6* in the CT group. Scale bar for **(A)**, **(C)**, **(E)** = 200 μm, and that for **(B)**, **(D)** = 500 μm. The black arrowheads labeled in the figure show the hybridization signal.

Table S1. Primers used in qRT-PCR.

Primer names	Sequences
U6-F	ACAGAGAAGATTAGCATGGCC
U6-R	GACCAATTCTCGATTTGTGCG
Lbr-miR171b-F	TGATTGAGCCGTGCCAATATC
Universal Reverse Primer	CTCAACTGGTGTCGTGGAGTC
18S rRNA-F	CCTGAGAAACGGCTACCACAT
18S rRNA-R	CACCAGACTTGCCCTCCA
LbrSCL6-F	ATGCCCTTCAATCTTCAACTGT
LbrSCL6-R	TCAGCACCTCCACGCTGA

Table S2. Combinations of LbrSCL6 and LbrWOX4 bimolecular fluorescence complementation assays. 1–3 are negative controls, and 4 is the test group.

Serial number	ENN-linked genes	ECN-linked genes
1	None	None
2	*LbrSCL6*	None
3	None	*LbrWOX4*
4	*LbrSCL6*	*LbrWOX4*

Table S3. LbrSCL6 and LbrWOX4 yeast two-hybrid test combinations. 1–3 are negative controls, and 4 is the test group.

Serial number	pNC-GBKT7 linked genes	pNC-GADT7 linked genes
1	None	None
2	*LbrSCL6*	None
3	None	*LbrWOX4*
4	*LbrSCL6*	*LbrWOX4*
